# The analysis of infrared high-speed motion capture system on motion aesthetics of aerobics athletes under biomechanics analysis

**DOI:** 10.1371/journal.pone.0286313

**Published:** 2023-05-25

**Authors:** Yaoyu Qiu, Yingrong Guan, Shuang Liu

**Affiliations:** 1 School of Sport, Shangrao Normal University, Shangrao, China; 2 College of Physical Education, Jinggangshan University, Ji’an, China; Ningbo University, CHINA

## Abstract

This paper uses an infrared high-speed motion capture system based on deep learning to analyze difficult movements, which helps aerobics athletes master difficult movements more accurately. Firstly, changes in joint angle, speed of movement, and ground pressure are used to analyze the impact and role of motion fluency and completion based on a biomechanical perspective. Moreover, based on the existing infrared high-speed motion capture systems, the Restricted Boltzmann Machine (RBM) model is introduced to construct an unsupervised similarity framework model. Next, the motion data is reorganized based on three-dimensional information to adapt to the model’s input. Then, the framework performs similar frame matching to obtain a set of candidate frames that can be used as motion graph nodes. After the infrared high-speed motion capture system and inertial sensors are simultaneously applied to subjects, the multi-correlation coefficients (CMC) values of the hip, knee, and ankle angles are 0.94 ± 0.06, 0.98 ± 0.01, and 0.87 ± 0.09, respectively. The two systems show a high degree of correlation in the measurement results, and the knee joint is the most significant correlation. Finally, a motion graph is constructed to control its trajectory and adjust its motion pattern. The infrared high-speed motion capture system optimized for deep learning can extract features from human bone data and capture motion more accurately, helping trainers to fully understand difficult movements.

## 1. Introduction

The skeletal system comprises many joints that must constantly respond to the human need for movement throughout the day [1]. The bones and muscles of the human body use a complex system to transmit external and internal stresses and loads. In recent years, biomechanics (the name for the study of biological structural systems) has developed rapidly. Medical researchers, biomechanical engineers, and laboratory technicians communicate on applying tests and measurements [2]. Recent research projects include using strain gauges to design prototypes of artificial composites to replace the joints that humans are born with [3]. Based on advanced sensor technology and a variometer, the experiment measures the pressure generated in the blood by the forces transmitted by various parts of the bone structure under various motion states.

In traditional sports training, the athletes master the movements under the guidance of experienced coaches. Infrared motion capture technology provides powerful data support for traditional empirical training, making the process more efficient [4]. By capturing and analyzing athletes’ data, coaches can more accurately identify differences and guide students to correct and optimize. The parameters of human functional movement are important indexes to evaluate the corresponding movements such as walking, running, jumping, and squatting. Based on three-dimensional (3D) key points and bones, ordinary cameras achieve full-body driving of virtual images. The camera can track even if the character has significant movement changes, occlusion, turning, and other situations [5]. As an analytical instrument used in the field of sports science, the infrared motion capture system uses a high-performance infrared lens to capture passive luminous marking points to construct a 3D movement acquisition and analysis system. In addition to sports motion analysis, the infrared motion capture system is widely used in gait rehabilitation, motion analysis, robots, drones, movie animation, game production, virtual simulation, and other fields [6].

In sports, aerobics is often accompanied by music. The choreography and completion of difficult movements determine the level of sports aerobics [7]. Athletes tend to challenge the world’s highest-scoring obstacle, Class C, which is over 160. The difficulty of the action is analyzed and evaluated by selecting relevant parameters, such as the hip joint angle, the knee joint angle, and the plantar pressure distribution. In the capture of aerobics movements, precision equipment is used to record the natural biological movements. Motion capture technology based on video recording converts the movement into computer data. This technology has been extensively used in animation production, sports training, ergonomics, and other fields [8]. The best way to capture motion using a single camera is to optimize the parameters of the 3D mannequin so that the secondary projection matches the measurement results in the video [9]. In this process, neural network models learn to map images into corresponding 3D mesh sequences. The deep learning models demonstrate superb capabilities in supervising large-scale annotated data sets. With the rapid development and practical application of motion capture technology, many realistic 3D human motion data have been accumulated. Therefore, effective semantic discrimination of motion capture data has important application value for data reuse and subsequent motion editing, retrieval, and simulation.

Nowadays, difficult movements are the key to winning competitive aerobics, which benefits from the mature development of this sport and the improvement of its competitiveness [10]. The difficult movements in aerobics are analyzed based on biomechanics to help trainers comprehensively understand the essentials of movements. As a typical representative of deep learning models, RBM has richly distributed hidden states. This model can perform concise and effective inference and has made breakthrough progress in multiple fields. Firstly, the advantages of infrared high-speed motion capture systems are explored. Then, a spatiotemporal feature extraction model is established using a deep learning network for the motion sequence of human bones. Based on the RBM model’s motion capture and behavior recognition, bone prior information helps to reconstruct the hidden space motion posture feature. Finally, the transition frame suitable for constructing the motion graph node is obtained to achieve more precise motion capture. Section 2 elaborates on the research status of the infrared high-speed motion capture system. Section 3 introduces the RBM model based on deep learning. An unsupervised similar frame model is established to detect the connection between adjacent joints and achieve accurate motion capture. Section 4 evaluates the performance of the ConvRBM model through simulation experiments. It proves that the model can more accurately extract the characteristics of human bone data and complete motion capture. The innovation of this research is that the constructed semi-supervised combination model extracts features. Then, its effectiveness is verified by the high-level discriminant model. Compared to traditional classification models, the constructed semi-supervised composite model utilizes generation capabilities to extract features. This approach is effective in classification tasks.

## 2. Research progress of infrared high-speed motion capture technology

Infrared motion capture technology can provide powerful data support for traditional experience-based training, making the training process more efficient. Existing research has confirmed that capturing and analyzing the movement data of the world’s top athletes can more accurately compare and judge the gaps in training movement. Therefore, the athletes can be guided to optimize their movements. Yu et al. (2017) [11] pointed out that infrared target tracking is important in surveillance videos. Combining infrared target feature representation with online structure learning-based detection and tracking methods is applied to the tracker to achieve high accuracy and robustness for infrared sequences. Naeemabadi et al. (2018) [12] pointed out that the motion capture system may interfere with the sensor and affect the sensor’s performance in tracking bones. Therefore, the human body model was used to study the influence of the motion capture system on the Microsoft Kinect v2 skeleton algorithm. The motion capture system had a devastating effect on the Microsoft Kinect v2 skeleton tracking algorithm.

Akula et al. [13] focused on the development of automatic, minimally intrusive, and privacy-protecting systems. In order to improve the robustness of the system, a supervised convolutional neural network architecture with two convolutional layers is designed to classify six action classes. On the complex test data selected manually, the classification accuracy of the system reaches 87.44%. Infrared imaging can achieve human gesture recognition in complex environments compared to visible spectrum cameras. Geng and Yin (2020) [14] pointed out a model to detect human gestures based on the improved YOLO-V3 network. The network can enhance the reuse of features and improve network performance based on the images in the second input channel.

Currently, the infrared high-speed motion capture system research mainly focuses on the performance development and stability optimization of the capture system. There is no research on the motion capture system’s actual application and function development. Here, the RBM model is introduced and applied to the athlete’s motion capture to help athletes master difficult movements more accurately.

## 3. Motion capture and analysis based on deep learning

### 3.1 Difficulty and aesthetics of aerobics

Sports biomechanics plays an important role in sports development, especially in technical analysis, training, design and improvement of sports equipment, and prevention of athlete injuries. The research of sports biomechanics provides support for the development of rehabilitation engineering. The demand and progress of rehabilitation engineering have promoted the research and development of sports biomechanics. The rehabilitation of sports injuries depends on the comprehensive application of biology, sports means, and mechanics. Sports biomechanics can provide a complete perspective. The high-difficulty jumping movements in sports aerobics have five characteristics. 1. The entire movement is completed in the air. There are one or more aerial movements. 3. The duration of air motion during the entire air process is long. The degree of rotation in the air determines the score of the same difficult action. There are four types of landing movements: one foot, two feet, push-ups (including push-ups, one arm push-ups, and Vincent push-ups), and splits (including horizontal and vertical) [15].

**Fig 1:**
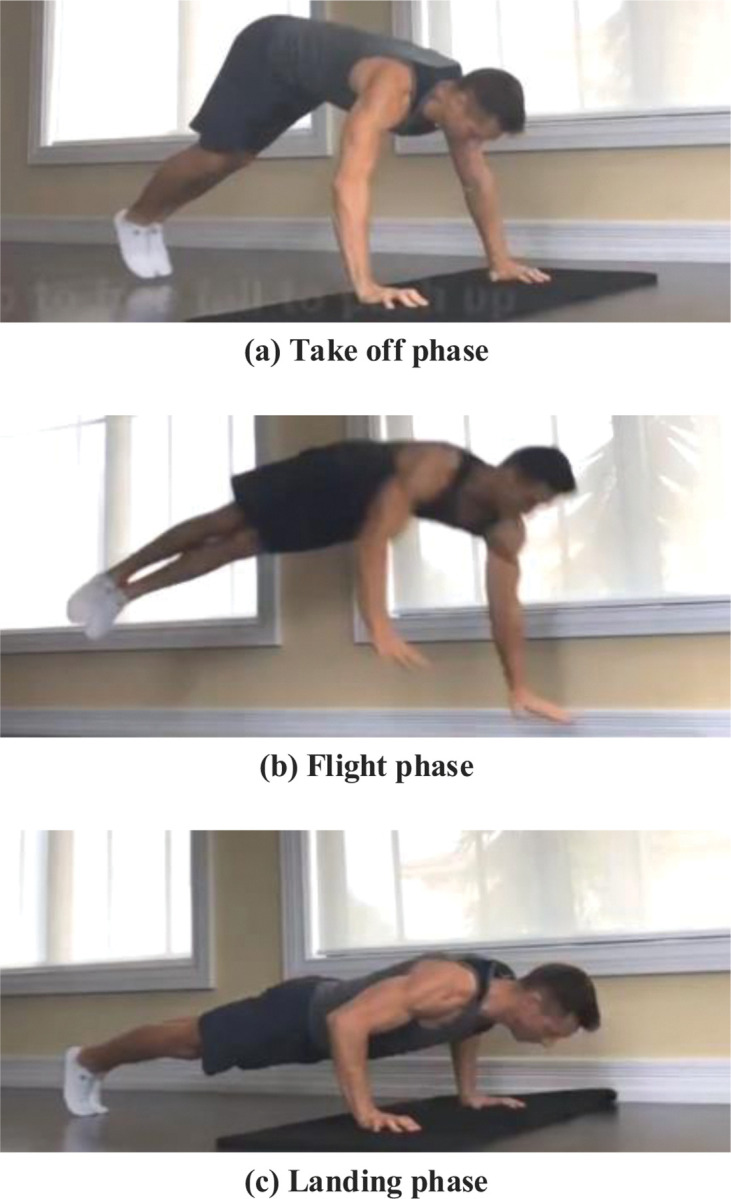
Three phases of jumping movements in sports aerobics.

The completion of difficult jumping movements can be divided into three phases: the take-off, the flight, and the landing, as shown in Fig 1. In the take-off phase, the three factors that determine the difficulty of jumping are the height, time, and torque of the jump, which are reflected in the flight phase. Takeoff preparation is an integral part of the jump process as it prepares for the flight phase. A reasonable take-off is a prerequisite for a perfect flight phase, influencing the height and time in the air.

Mechanical analysis of the take-off stage of a single type of movement. The take-off phase requires instantaneous maximum upward or diagonal speed to prepare for sufficient height and time. The athlete must stretch all joints quickly when leaving the ground, including the toe, ankle, knee, and hip joints in sequence and the coordinated upper limb joints such as the shoulder and elbow. Acceleration of each joint upward produces a reaction force on the force application site, which acts on the ground through muscle contraction, increasing take-off force. These preparations enable the athlete’s body to achieve sufficient vertical speed when leaving the ground [16, 17]. In addition, the full extension of the joints lifts the relative position of the center of gravity, shortening the take-off time.

A mechanical analysis of the take-off stage of complex movements. Complex movements put higher requirements on athletes, often by turning in the air. At this time, the support reaction force during the take-off phase must be eccentric. Hence, it is necessary to control the take-off angle and direction, to reduce the forward component and speed, thereby obtaining a greater tangent component. In the take-off phase, the arms and the free leg should rotate and swing upward, and the body will rotate slightly accordingly. However, the center of gravity should not rush forward too much, which will cause a loss of speed and rotation energy [18–20].

As sports aerobics march forward continuously, more and more athletes challenge multiple difficult movements. The quick and powerful take-off and the resulting rotational energy guarantee the completion of the take-off stage. When the flight height and flight time are fixed, the turning torque determines the completion of the turning. During the entire flight phase, athletes rely on the muscles and joints of their limbs to complete the movement. The maintenance of the aerial shape is particularly important in the flight phase, and it will directly affect the evaluation of the referee. Therefore, athletes should have quick take-off and landing but slow down to change postures for better visual effects [21].

From a biomechanical perspective, when an athlete is about to land, the body’s internal force is essential for the control and completion of the action. Therefore, athletes should strengthen their theoretical study of difficult jumping movements to familiarize themselves with "standard norms.". In training, athletes try to use their own will to control and perfect the movement.

### 3.2 Infrared high-speed motion capture system

The infrared high-speed motion capture system uses a high-speed infrared camera to capture passive luminous marker points and constructs a 3D motion acquisition and analysis system. Due to the extremely rapid changes in motion trajectory, it is difficult for humans to recognize every subtle movement with the naked eye [22, 23]. During whole-body exercises, at most, more than 400 muscles will work simultaneously. It is very challenging for students to quickly judge the biomechanics involved in each small movement. Motion capture technology visualizes sports movements so students can observe them more clearly and obtain data reference. This technology could be a huge help to sports research and education. The motion capture system composed of cameras can record the motion trajectory of multiple passive marker points. As a result, it can capture and record the whole body’s motion trajectory and select the marked parts according to the needs. More importantly, it hardly loses any information [24–26]. The principle of an optical motion capture system is that when a camera emits infrared light into an active area, it encounters passive marker points that are reflected and recognized by an infrared light receiver. High resolution and sampling frequency can be achieved simultaneously in infrared high-speed motion capture systems, especially sensors, resulting in good 3D motion capture effects and strong functionality. The system is widely used in sports training, ergonomics research, biomechanics research, and other fields.

**Fig 2:**
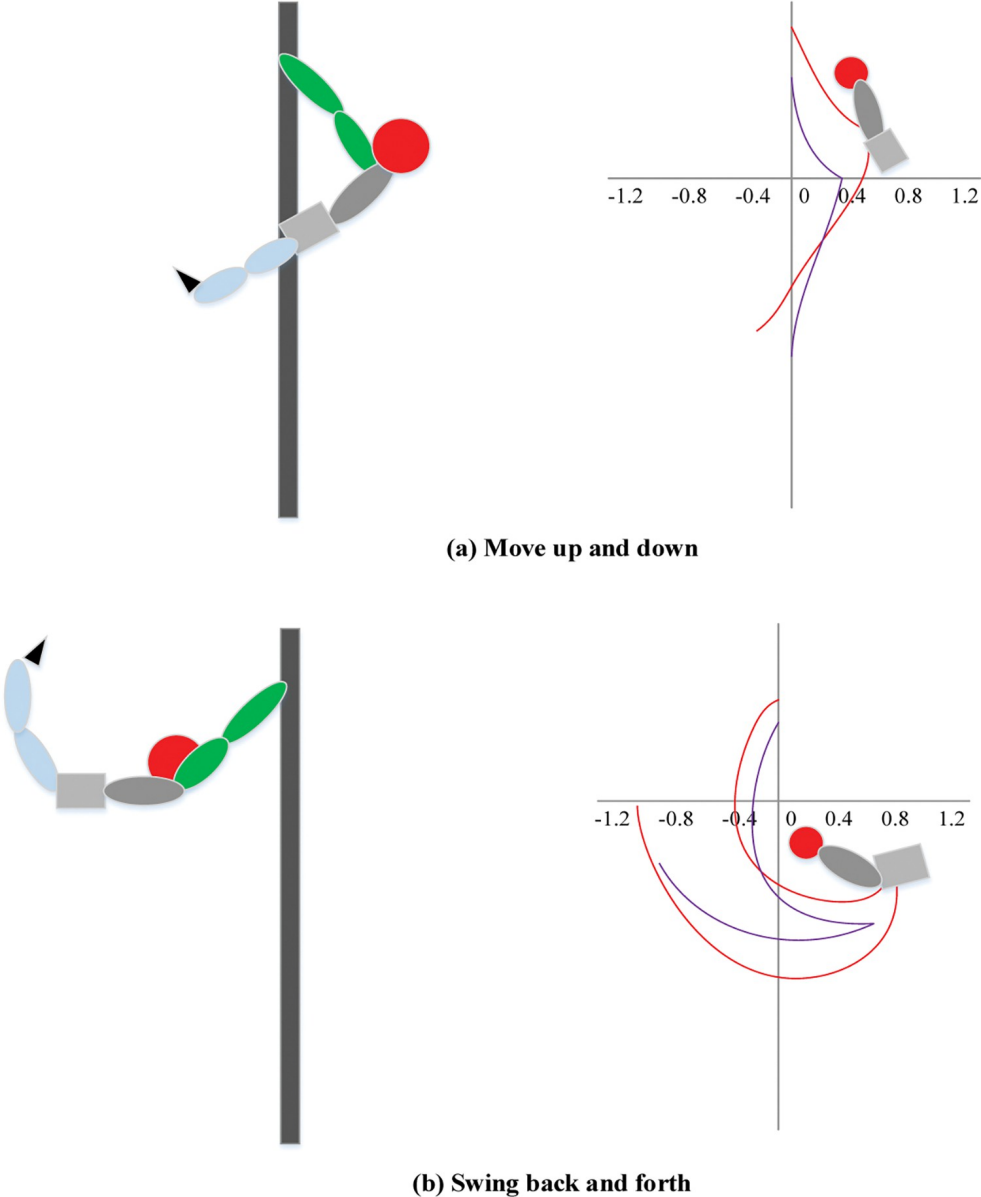
Infrared high-speed motion capture of horizontal bar motion.

The Opti Track motion capture system is generally regarded as the "gold standard" for motion trajectory capture because it records motion trajectories at the sub-millimeter level. The horizontal bar movement is an example to analyze the movement capture methods of moving up and down and swinging back and forth, as shown in Fig 2. The movement of the pelvis depends on the angle changes of the hip joints.

**Fig 3:**
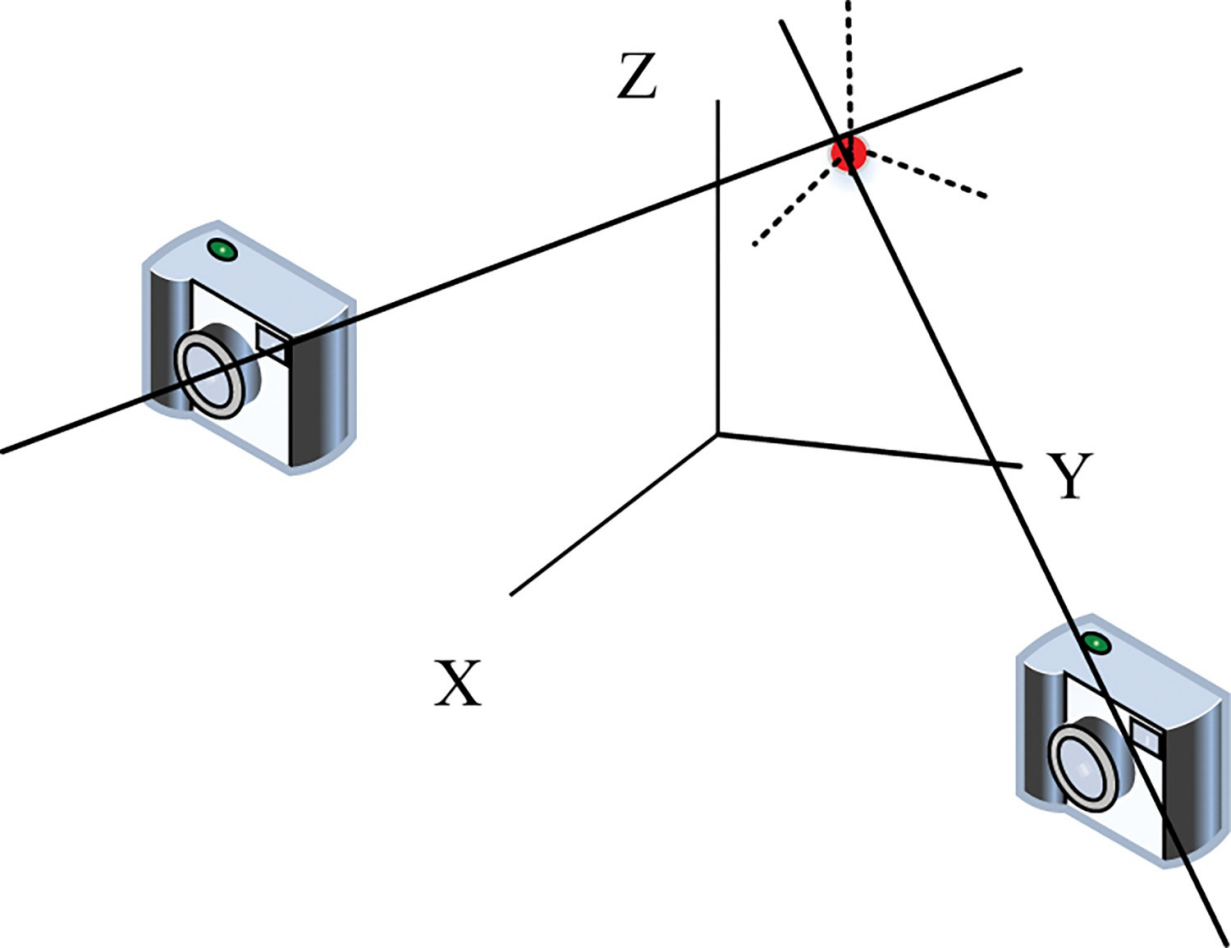
3D reconstruction of the motion capture system.

The infrared motion capture system requires multiple cameras. It uses a 16-megapixel camera to fuse video animation with space to obtain 3D coordinates for landmarks. After the collected motion data is processed by related software, the complete 3D bone structure is displayed. Calibration refers to two cameras capturing the same point simultaneously and obtaining corresponding coordinates after determining camera parameters, as shown in Fig 3.

In actual 3D reconstruction, marking a certain shape as a point will produce errors. When the same landmark is captured by multiple cameras spontaneously, there will be errors in the 3D reconstruction data between each other. If the real position of the marker point is expressed as *P*_*r*_, the real imaging points are *R*_1_, *R*_2_, the recognized imaging points are *I*_1_, *I*_2_. *δu* and *δv* are the non-linear distortions, as shown in Eqs (1)–(4).


$$u-\Delta u-\frac{L_{1}x+L_{2}y+L_{3}z+L_{4}}{L_{9}x+L_{10}y+L_{11}z+1}=\delta u$$ (1)

$$v-\Delta v-\frac{L_{5}x+L_{6}y+L_{7}z+L_{8}}{L_{9}x+L_{10}y+L_{11}z+1}=\delta v$$ (2)

$$\overset{c}{\underset{i=1}{\sum }}\delta U_{i}=0$$ (3)

$$\overset{c}{\underset{i=1}{\sum }}\delta V_{i}=0$$ (4)

Where *c* represents the number of cameras, Δ*u* and Δ*v* represent the optical errors, and the parameters *L*_1_~*L*_11_ represent the relationship between the image and the object space coordinate system. The coordinates of the object space (*x*,*y*,*z*) can be calculated according to the coordinates of the image space (*u*,*v*).

The basic principle of 3D reconstruction optimization is to minimize the value of uncertainty *ε*, as shown in Eq (5).


$$\varepsilon =\sqrt{\frac{1}{c}\overset{c}{\underset{i=1}{\sum }}(\delta U_{i}^{2}+\delta V_{i}^{2})}$$ (5)

The uncertainty of the single mark point *ε*_*a*_ and the multi-mark points *ε*_*b*_ are calculated as shown in Eqs (6) and (7).


$$\varepsilon_{a}=\sqrt{\frac{1}{cf}}\cdot \overset{f}{\underset{j=1}{\sum }}\overset{c}{\underset{i=1}{\sum }}(\delta U_{ij}^{2}+\delta V_{ij}^{2})$$ (6)

$$\varepsilon_{b}=\sqrt{\frac{1}{cfp}\cdot \overset{p}{\underset{k=1}{\sum }}\overset{f}{\underset{j=1}{\sum }}\overset{c}{\underset{i=1}{\sum }}(\delta U_{ijk}^{2}+\delta V_{ijk}^{2})}$$ (7)

Where *f* indicates the number of frames and *p* indicates the number of mark points.

First, the parameters of the camera pinhole model are calculated according to the principle of small distortion in the middle area of the image. Then, the initial value of the distortion parameter is calculated by introducing the global view. When the verticality of the optical axis and the object surface is high, the focal length of the pinhole model lens and the components of the translation matrix are highly correlated, and it is difficult to calibrate accurately at one time. Therefore, the initial value of the distortion parameter needs to be substituted into the model, and other parameters that have little influence on the calculation accuracy are assumed to be fixed values. A two-step iterative method is used to approximate the distortion parameter’s exact solution gradually.

When the infrared high-speed motion capture system is applied, a marker point reflecting infrared light should first be set on the measured object. The most accurate measurement of bone motion can be obtained by setting markers on the bone pins [27]. However, the method is not often used because it is invasive and may injure the human body. Another method is to set the mark point on the body surface corresponding to the anatomical bony mark point, which is easy to operate. However, the movement of skin, muscle, and other soft tissue will change the location of the mark point. After all, the human body is different from a rigid body. Especially in momentary collision motions, the location of the mark point changes greatly. It is more scientific to set the mark point on a rigid structure attached to the human body. However, the setting of the marker points does not affect the movement and can be recognized by at least two cameras in the dynamic test [28–30].

As per Helen Hayes’ method of setting the marker points, a coordinate system of the pelvis, calf, thigh, and ankle is established. In the pelvis, the infrared reflective points are pasted on the sacrum, the anterior superior iliac spine of both sides, and the highest point of the iliac spine. In the thigh, the infrared reflective points are pasted on the anterior part of the thigh and the medial and lateral condyles of the femur. In the calf, the infrared reflective points are pasted on the anterior part of the calf, fibula lateral malleolus, and tibia medial malleolus. The infrared reflective points are pasted in the feet on the fourth metatarsal, lateral malleolus, and heel.

### 3.3 Motion capture model based on deep learning

**Fig 4:**
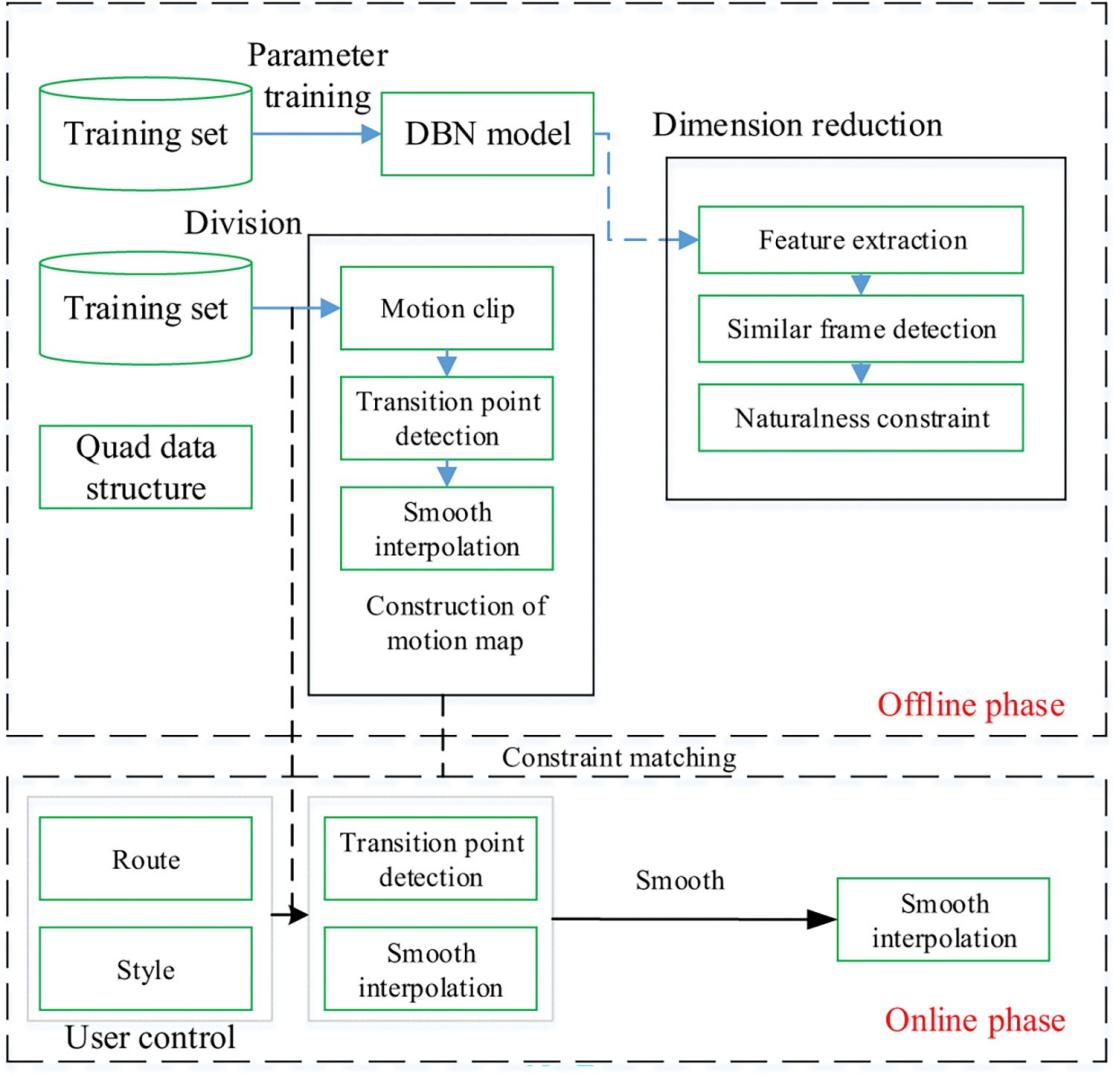
The construction of motion capture.

Based on the feature extraction of deep learning and the motion capture that supports flexible switching between multiple sports styles, an unsupervised deep learning model is proposed to extract the spatio-temporal features of motion capture sequences. The declination-based segmentation method is used to perform motion capture. In the matching process, retrieval efficiency is lifted. The construction of motion capture is shown in Fig 4. In the offline stage, much sample data is used to train the CRBM model. Therefore, it can better detect transition points in the motion map for the user data set. In the online stage, segment segmentation, and style retrieval are performed based on the input trajectory. Online input can match offline motion graphs under constraints based on the quaternion structure of the deflection information.

Motion capture files contain rotation Angle data for each joint in the body. Therefore, the first derivative of different frame intervals is used to calculate the instantaneous angular velocity of the joint, eliminating the influence of directional information on the time axis. The 3D coordinates of each joint are calculated based on the hierarchical relationship between joints and the rotational Euler angle in forward kinematics. Then, reference points such as the center of gravity of the human body or the root node of each frame are selected to calculate the relative positions of the remaining joints and the reference points. Finally, the impact of different absolute positions of the same posture on the recognition effect is eliminated.

**Fig 5:**
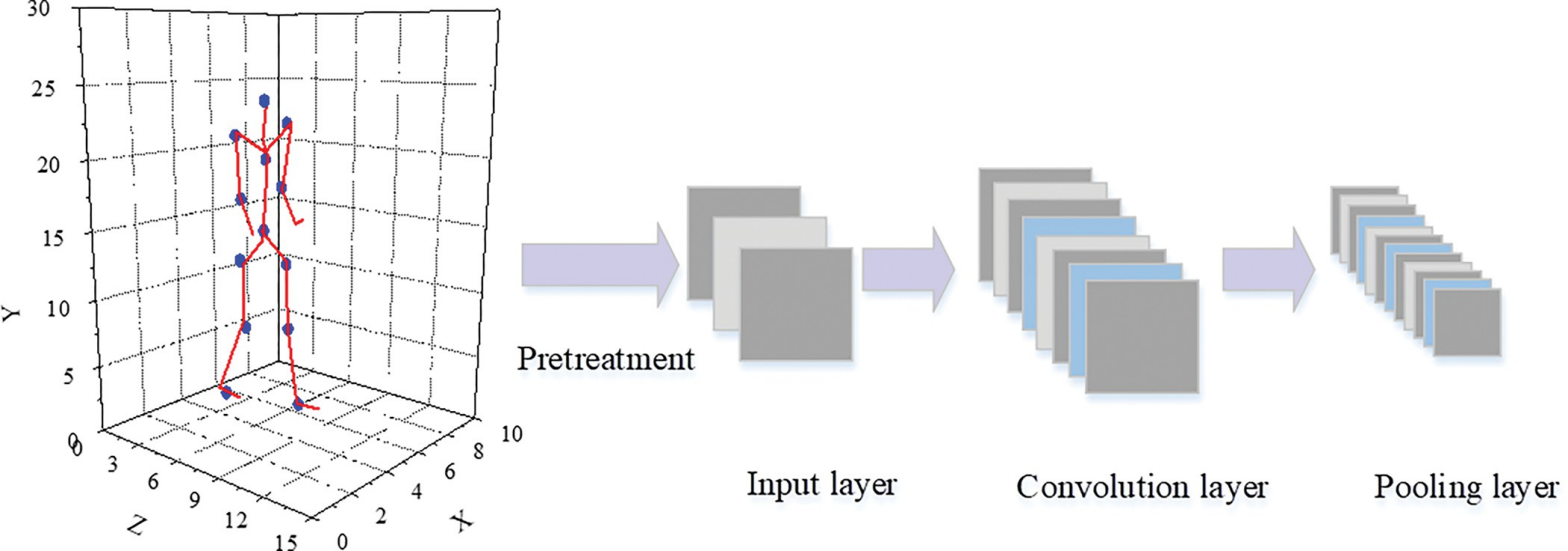
3D-ConvRBM model.

The 3D point cloud coordinates of the original bone structure are converted into a coordinate structure, and stored in three channels. In order to process human bone data using a deep neural network (DNN), the human body needs to be divided into five parts: trunk, left upper limb, left lower limb, right upper limb, and right lower limb. Each part is preprocessed separately, but this method ignores the local bone and joint information [31, 32]. The RBM model can extract static frame features, and the ConvRBM is proposed for unsupervised learning. As one of the representative deep learning models, the RBM model can extract static frame features. CRBM model can be established by adding an autoregressive model to the input layer to obtain temporal feature information with contextual semantic scenarios. The RBM can reflect the generation process of the target object and the similarity between objects through the energy function and the activation state of the hidden layer neurons. The ConvRBM is used to extract spatio-temporal features of 3D data and process the temporal dependency of the internal spatial structure of the bones in each frame of the motion capture data [33, 34]. The structure of the 3D-ConvRBM model is shown in Fig 5. Unsupervised learning is carried out using 3D-ConvRBM and deep belief networks. The 96-dimensional point cloud data is mapped into a low-dimensional feature space, and the candidate transition frame group is filtered based on a preset threshold.

In the 3D-ConvRBM model, neurons in each sensory area and convolutional layer can form a miniature RBM structure. The motion graph is the same as the graph model, composed of nodes and edges. Each node comprises moving frames with similar postures, and each edge contains intermediate transition frame sequence and similarity information. Both of them are used to assist in the selection of a transition path. In order to transition from a certain movement segment to another style, if there are multiple paths, the transition edge with the highest similarity is selected to ensure the rationality of natural transition. The 3D-ConvRBM model combines the advantages of supervised learning and test time optimization. Supervise learning to initialize parameters at the correct time and ensure good posture and surface initialization during the test process without manual operation. Self-supervision through backpropagation with rendering can adapt the model to the test data and provide a better fit than training a fixed model. Compared with the traditional method of constructing a motion graph, the key posture extracted by deep learning is more conducive to creating a natural motion transition sequence while flexibly controlling the motion.

### 3.4 Experimental design

The experiment aims to test the motion capture effects of an infrared high-speed motion capture system and the inertial sensor. Fifteen young males, aged 22.5–25 years old, were selected as research subjects, with a height between 175.3±7.7cm and a weight between 73.2±7.2kg. The infrared motion capture system is used to calibrate the venue space. The subject walks on the data collection site in the most comfortable gait, with an infrared reflective point pasted on the left lower limb, and wears an inertial sensor. There is five sets of data collected. The Noraxon Biomechanical Sensor Motion Capture System is a 3D motion system containing 16 sensors. The MR3 software of the system uses the collected information to build a steel body model of the human to calculate the joint angle. The infrared high-speed motion capture system comprises a computer, eight infrared cameras, and infrared reflective points. The 2D coordinates of the image can be obtained using one camera, while the 3D coordinates need to be obtained using two or more serial cameras to measure spontaneously. In order to verify the performance of the motion capture algorithm based on deep learning, three styles of motion data are extracted from the Carnegie Mellon University (CMU) database: walking, running and jumping. The amount of data used allows the motion capture model to extract the motion features of multiple styles.

### 3.5 Experimental environment and parameter settings

This experiment uses Matlab2015b software, the processor of the operating environment is Intel Core i3, and the operating memory of the PC is 8G. The data collected by the inertial sensor is processed by MR3.10 software, and the data collected by the infrared high-speed motion capture system is processed by Cortex software. On a computer configured with an Intel Core i7-6700 processor and 16G of memory, the algorithm is run based on Matlab 2018b, with a processing speed of approximately 30 frames per second. This configuration meets the speed requirements of the motion capture system. The multi-correlation coefficient (CMC) between calibrations is used to evaluate the differences in the motion capture system between different calibrations. The error between calibrations is used to evaluate the angular difference of the measured values of the motion capture system. The simulation platform of this study conforms to the specifications.

## 4. Results and discussion

### 4.1 Measurement results of the infrared high-speed motion capture system

**Fig 6:**
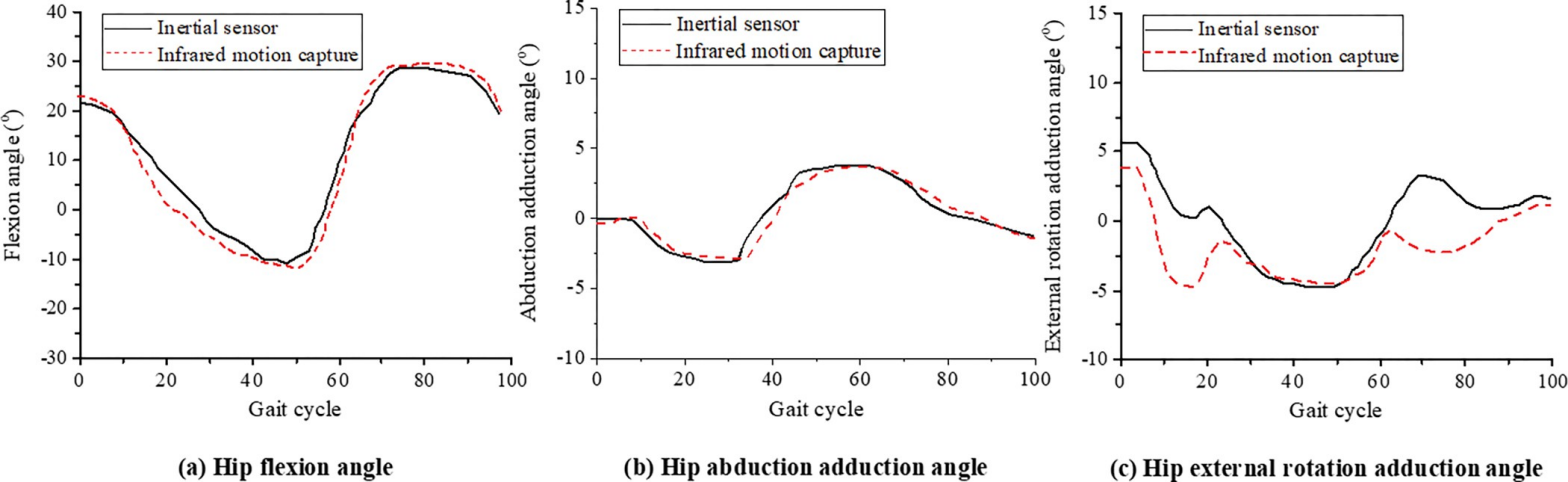
The mean curve of the hip joint angle during walking.

**Fig 7:**
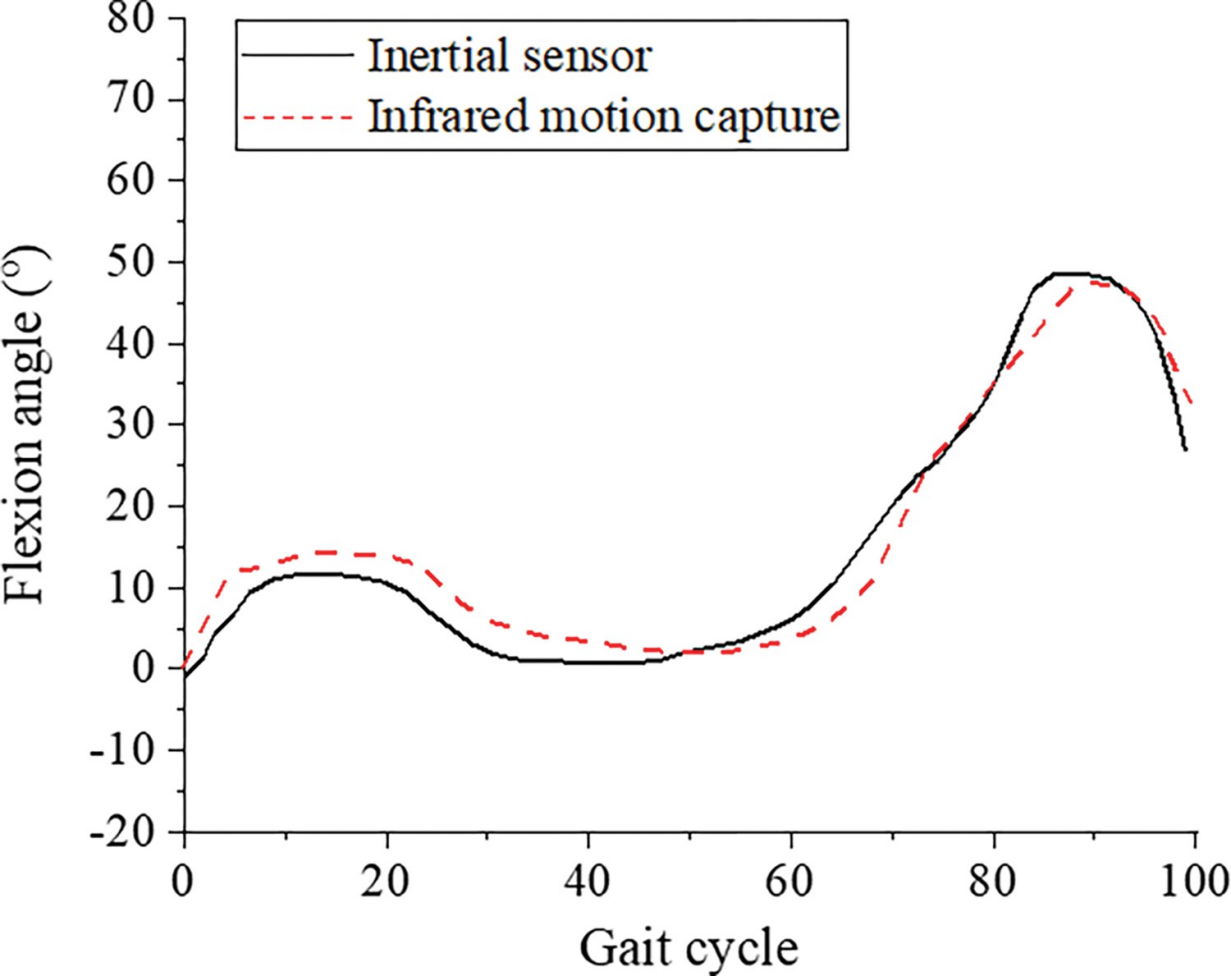
The mean curve of the knee joint angle during walking.

**Fig 8:**
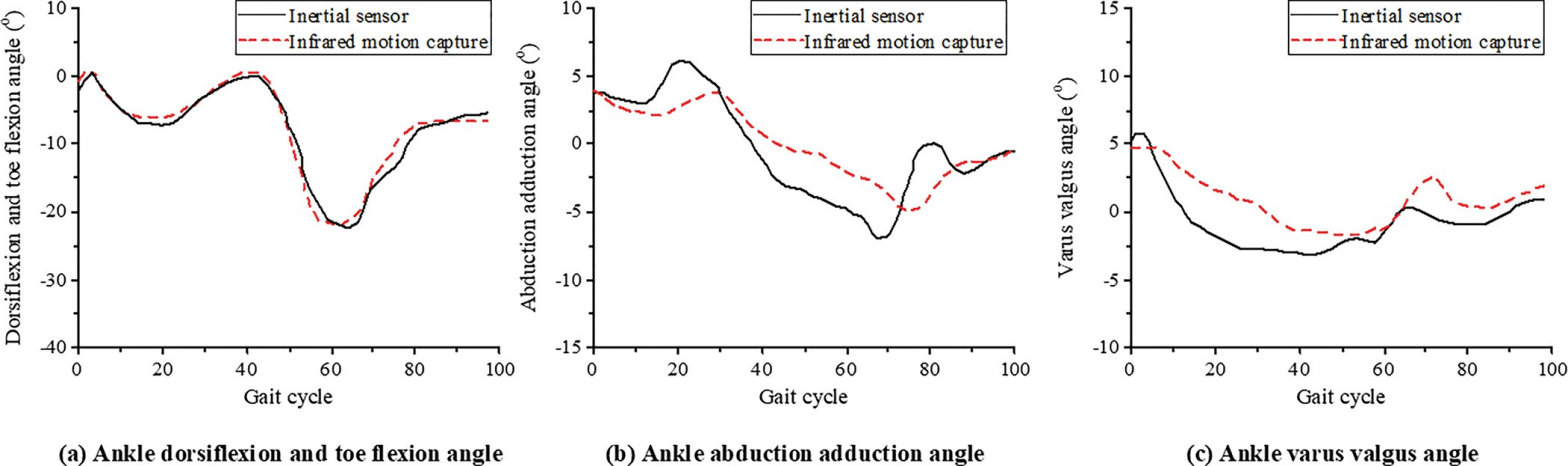
The mean curve of ankle joint angle during walking.

During walking, the change curves of joint angles of the lower limb hip joint, knee joint, and ankle joint collected by the infrared high-speed motion capture system and inertial sensors are shown in Figs 6–8. In order to compare the joint angle curves measured by the two motion capture systems, the joint angle curves of the infrared high-speed motion capture system are translated up and down to make it’s mean equal to that of the inertial sensor. The graph after MeanShift can more intuitively reflect the correlation and difference between the two motion capture systems.

A CMC value closer to 1 indicates closer measured values and stronger similarity of the two systems. A CMC value closer to 0 indicates greater differences between the two systems. The CMC values of the hip, knee, and ankle joint angles in the flexion extension are 0.94±0.06, 0.98±0.01, and 0.87±0.09, respectively. The two systems show a high correlation in the measurement results, especially the knee joint correlation between the two systems. During walking, the CMC value of the hip joint abduction-adduction angle is 0.70, indicating that the two systems are fairly correlated. The CMC values of the hip joint angle are lower than 0.41 in both abduction-adduction and varus-valgus, indicating that the two systems are poorly correlated.

### 4.2 Detection of motion transition point based on ConvRBM

**Fig 9:**
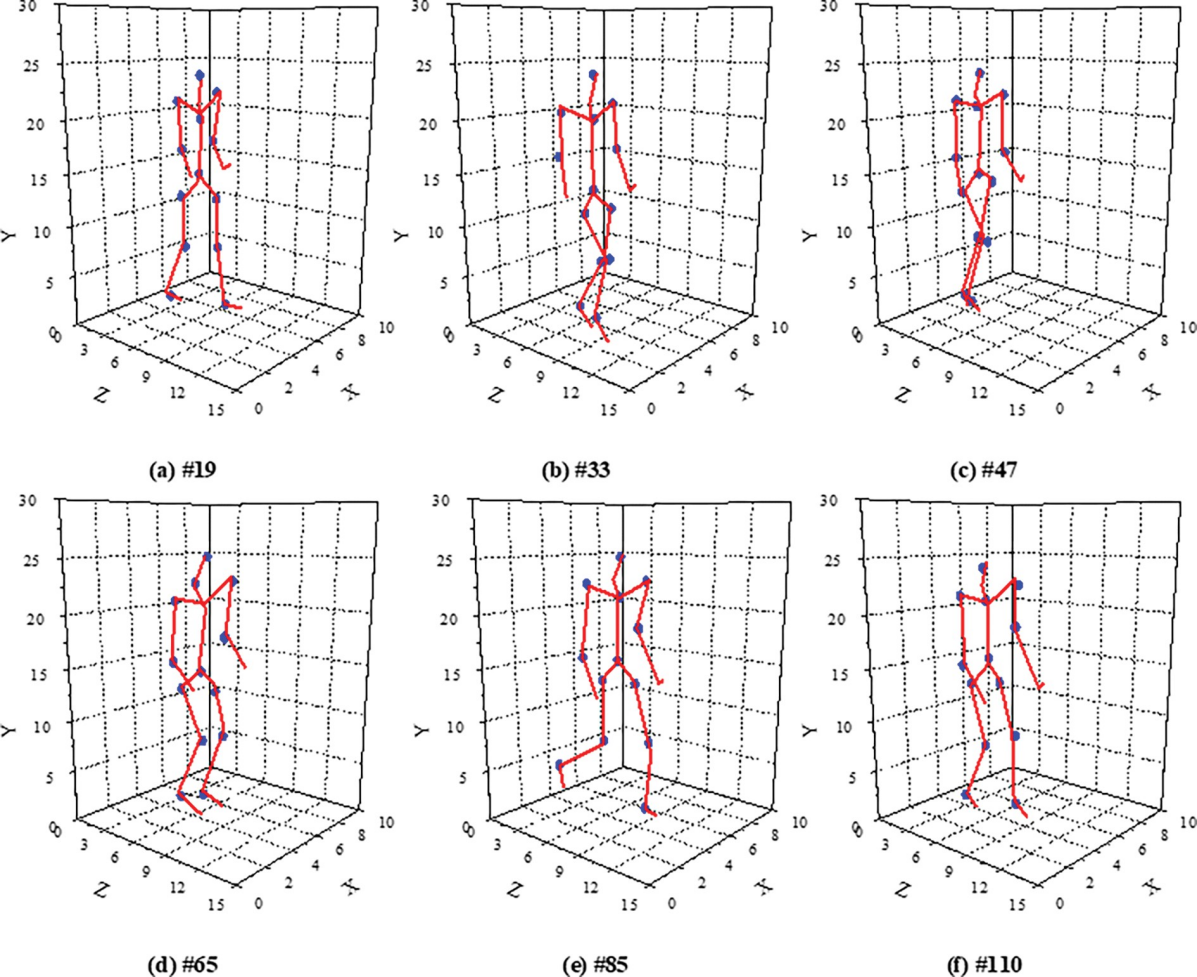
Search results of similar motion frames based on the ConvRBM.

**Fig 10:**
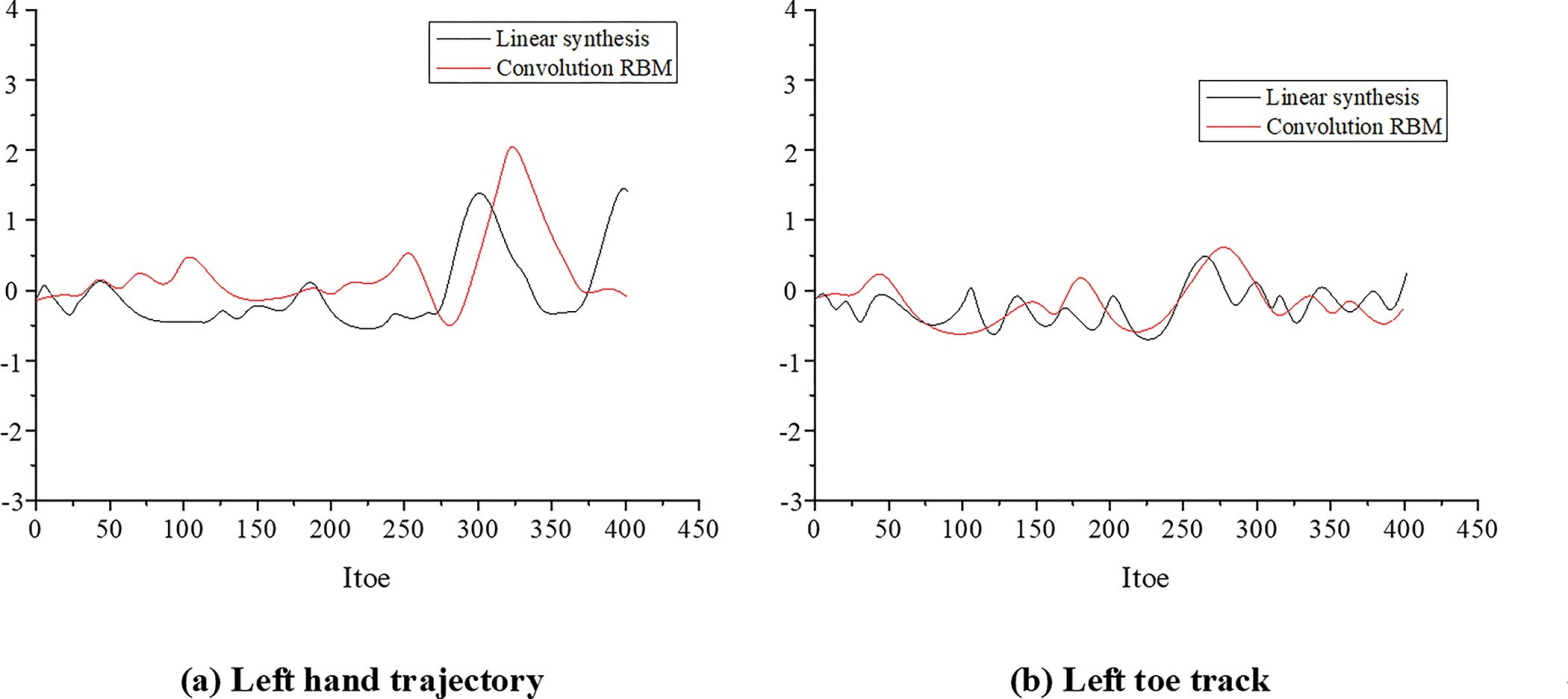
The projection of sports-style conversion on the "yz" plane.

During the movement, the transition frame segment is used for the conversion of sports style, and it follows that the quality of the transition frame will affect the overall continuity of the movement sequence. Similar frames can be retrieved by mapping the motion to a 2D feature space. The search results of similar frames are analyzed in the 19th, 33td, 47th, 65th, 85th, and 110th frames in the running motion data "15_32.amc", as shown in Fig 9. Thirty frames are selected as the window length of the linear synthesis algorithm, with the first and last frames of the motion segment to be spliced as the start and end frames of the mixed segment, respectively. When the method based on ConvRBM is used to detect motion transition points, once two similar frames are detected, the interpolation algorithm can be used to generate intermediate frames based on the calculated transition length. With the transition of "walking to running" as an example, the movement trajectories of the two end effectors of the left hand and left toe are extracted, respectively. The projection on the "yz" plane is shown in Fig 10. The body movement is usually accompanied by slight body shaking, affecting the data quality during the motion capture process. In the motion graph sequence constructed with the original motion segment, different degrees of noise are noted in the joint trajectory.

### 4.3 Comparison of the recognition rate

**Fig 11:**
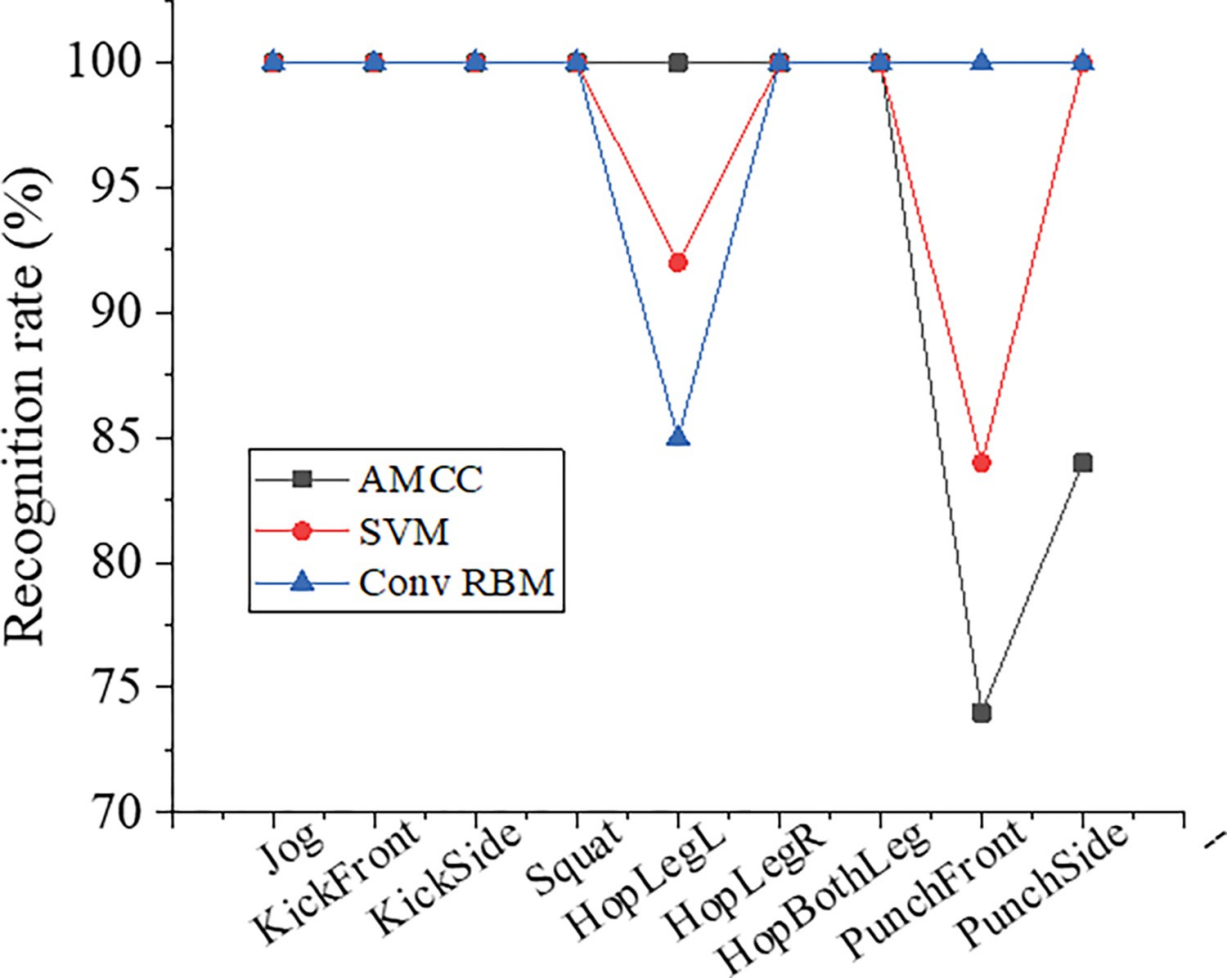
Comparison of the recognition rate by different algorithms.

Fig 11 shows the recognition rates of the three recognition algorithms. The convolutional RBM model can achieve good style classification results for simple motions. For simple sequences such as Jog, KickFront, and KickSide, its recognition rate reaches 100%. The adaptive motion codebook classifier (AMCC) and support vector machine (SVM) algorithms have a recognition rate below 70% for PunchFront with unobvious semantics. Those two algorithms focus on the spatial information of the joint human points that have a bigger influence on the movement style and ignore the timing information. This result is basically consistent with the literature results [35], verifying the reliability of the study. The depth model established needs only two sets of limited parameters to represent the sequences and only requires part of the training samples to learn. What’s more, it occupies a small storage space. Therefore, the algorithm in this study is suitable for learning and modeling large-data-volume motion sequences.

## 5. Conclusion

In sports aerobics, the difficult movements and the completion quality affect the scores. The infrared high-speed motion capture system enables the trainer to perceive the movement concretely because it can collect and analyze the kinematic data of difficult movements well. Hence, the infrared high-speed motion capture system analyzes difficult movements. Since machine learning theory has been gradually applied to the modeling of skeletal animation, it is applied in the motion capture system. Then, the spatio-temporal feature extraction model of bones is established with the ConvRBM. The motion data is reorganized according to the three-dimensional information and matched with similar frames. The candidate frame group is obtained and used as the motion graph node. Three styles of motion data walking, running, and jumping, are collected from the CMU database to test the performance of the motion capture algorithm based on deep learning. Under the motion capture structure based on ConvRBM, the motion graph is dynamic. The overall rotation is achieved based on the direction of motion of the current trajectory to avoid damaging the internal structural features of the original motion. The conclusions of this study promote the application of deep learning in infrared high-speed acquisition systems. However, the motion graphics are derived from the same motion video. In the future, research needs to test more sports styles further.

## Supporting information

S1 Data (XLSX)

## List of abbreviations

AMCC: adaptive motion codebook classifier
CMC: multi-correlation coefficients
CMU: Carnegie Mellon University
ConvRBM: convolutional RBM
DNN: deep neural networks
RBM: restricted Boltzmann machine
SVM: support vector machines
